# Healthcare waste management knowledge, attitudes and practices of laboratory workers at a regional hospital, Lesotho

**DOI:** 10.4102/ajlm.v13i1.2485

**Published:** 2024-12-06

**Authors:** Ts’aletseng M. Siimane, Motlatsi E. Nts’ihlele

**Affiliations:** 1 Department of Environmental Health, Faculty of Health Sciences, National University of Lesotho, Roma, Maseru, Lesotho

**Keywords:** healthcare waste management, knowledge, attitudes, practices, biosafety, biosecurity

## Abstract

**Background:**

Safe management of healthcare waste (HW) safeguards laboratory biosafety and biosecurity. Knowledge and attitudes influence HW practices, presenting a need for evidence of the current status.

**Objective:**

This study assessed the knowledge, attitudes and practice of laboratory workers towards waste management at a regional hospital laboratory in Lesotho.

**Methods:**

The study was conducted from March 2023 to June 2023 using a mixed-methods descriptive case study design. The entire population (*n* = 30) of technical and non-technical laboratory workers and generated waste were sampled. A structured questionnaire and an observational checklist were used to collect data. Waste generation was assessed by weighing and measuring waste volumes. Data were analysed using descriptive statistics.

**Results:**

All respondents (26/26; 100%) can define HW and (3/3) laboratory assistants (100%) gave correct responses for three questions, namely: risk associated with HW, waste container colour-coding, and disposal requirements. Knowledge on waste management responsibilities ranged between 0% (0/4) for cleaners and 54.5% (6/11) among laboratory technicians. Attitudes were mainly positive, and practices conformed in part to standard operating procedures. Infectious solid waste comprised 77% of solid HW, while 63% of chemical liquid waste emanated from the full blood count area.

**Conclusion:**

Knowledge exists among workers and attitudes are predominantly positive; however, some unsafe practices continue, thus knowledge is not fully translated to safe practices. Regular training and measuring and recording of HW were recommended.

**What this study adds:**

The study contributes understanding of the status of HW knowledge, attitudes and management practices, highlighting the need for compliance monitoring.

## Introduction

Healthcare waste (HW) is defined as all waste generated within healthcare facilities, research centres, and laboratories related to medical procedures.^1^ Medical laboratories, in particular, generate various categories of HW including general, infectious, pathological, sharps, and chemical waste. Healthcare waste generation rates are predominantly lower in developing countries than in developed countries, and a steady global increment of the HW production is observed worldwide, partly due to improved access to healthcare services.^2,3,4^ The risk posed by HW such as laboratory-acquired infections, injuries, toxicity, and cancers from carcinogenic chemical substances should be mitigated in the interest of biosafety. Equally important, poor practices may provide opportunity for threats to access pathogens and toxins, thus hampering biosecurity. Safe management requires a robust system with built capacities and the importance of staff knowledge, attitudes, and practice (KAP) cannot be overemphasised in such systems.

Developed countries deal with the fine-tuning of their already established systems, while developing countries are faced with the difficulties of setting up systems that work,^5^ and are burdened with huge national debt, lowering their possibility of investment in improved healthcare waste management (HWM) technologies and best practices. Developing countries need to establish affordable systems.^5^ Annual HW generation is predicted to increase in the coming years.^6^ African countries generate between 0.53 kg/bed/day and 1.53 kg/bed/day, while Asian countries show ranges between 0.5 kg/bed/day and 5.3 kg/bed/day. Developed countries in North America and Europe have generation rates of 1.7 kg/bed/day – 8.4 kg/bed/day.^7^ Among the factors influencing generation rates, higher gross domestic product and increased access to health services are associated with higher generation rates.^3^ The trends indicating an increase in waste generation imply laboratory personnel have more interactions with HW and thus will need thorough risk assessment followed by relevant mitigation and monitoring of HWM system performance. Adequate knowledge (and positive attitudes) about HW risks can go a long way in influencing the methods of handling waste, and the practice of safety and protecting public health and the environment.^8^

Various studies^3,9,10,11^ have indicated that generally there is an increased awareness among health professionals about the HW hazards and appropriate management techniques. An abundance of developing countries, however, still face inadequate knowledge and awareness as a result of inadequate training and a lack of safe systems for practising safe HWM.^12^ It remains important to note that there are still areas of lower to no knowledge, including the dangers of inhalation or skin exposure of chemicals, and reporting of incidents such as needle-stick injuries.^2^ Furthermore, even some developed countries have been facing challenges with regard to making the hospital employee’s behaviour and actions more sustainable. An intervention study has shown that a training programme brings significant increases in pre- and post-intervention scores among health workers.^13^ Mehta et al.^14^ stated that training has also been observed to bring about improved attitude and practice scores among the trained, compared to untrained, personnel.^14^ Other studies^15,16,17^ showed inadequate or low knowledge could not be ignored, since some^5^ associate low knowledge with risky practices. Laboratory workers are among the personnel with good knowledge regarding colour coding and waste segregation at source.^8^

In terms of attitudes, significant and positive correlations were found between knowledge and attitude, knowledge and practice, and practice and attitude.^18^ In addition, findings by some studies^14,19^ identified that more than 90% of the participants in their studies had a positive attitude towards HWM. It is imperative, therefore, that further studies correlate the attitudes to practice; however, adequate knowledge of HWM is one of the strategies that was found to impact positively on attitudes.^13^ With varying levels of investments made to improve HWM, a gamut of studies^8,20,21,22^ show unsatisfactory and unsafe practices which jeopardise biosafety and biosecurity. Countries should therefore aim at dealing with factors such as the high cost of training, lack of policies, failure of planning at national and facility levels, poor law enforcement from regulatory bodies, and lack of appropriate waste management responsibilities and utilities, which are barriers to safe and secure HWM practice.^5,23,24^

The management of HW has been of major concern because of potentially high risks to human health and the environment.^25^ Waste has historically not been managed safely; however, there have been positive strides in some countries such as Bahrain.^5^ Advancements in different countries have led to the development of policies, legislation, standards, and guidelines to mitigate the impact of HW, but there is a question of implementation which calls for analysis on the KAP of HWM systems.

Medical and diagnostic laboratories are mandated to maintain biosafety and biosecurity in their operations in an effort to align with national and international legislation and treaties. Lesotho developed the national HWM Policy (2010),^26^ Integrated Waste Management Strategy (2022),^27^ Hazardous (Healthcare) Waste Management Regulations (2012),^28^ and HWM Guidelines, Procedures and Tools (2012).^29^ Training is done at various intervals for cadres such as incinerator operators, environmental health practitioners, laboratory staff, hospital administrators, and others. All health facilities have access to incineration. Irrespective of these developments, 17% of nationally available incinerators are malfunctioning, and only 39% of health facilities were segregating according to the basic three-bin system. The majority of health facilities do not have HWM plans. Since the promulgation of the Lesotho Hazardous (Healthcare) Waste Management Regulations (2012),^28^ no such study has been published that interrogates the KAPs on HWM in Lesotho with a focus on the laboratory setting, especially following a series of trainings offered on the subject. This study therefore aimed to assess the KAP of laboratory staff in a regional hospital laboratory in Lesotho. To the best knowledge of the researchers, no such study has been published, and it is against this background that the study was undertaken. It is anticipated that the findings herein will go a long way toward informing HWM practices in the specified laboratory, and will serve as a benchmark for improving practice in other laboratories.

## Methods

### Ethical considerations

The study was subjected to review by the Institutional Review Board of the Faculty of Health Sciences, National University of University of Lesotho (NUL/EN/2023). Ethical clearance was also sought from the Ministry of Health’s Research Ethics Committee (ID55-2023). To access the study site, permission to conduct the study was received from the management of the hospital and the laboratory. Care was taken to gain written informed consent from participants. Privacy was ensured by obtaining written consent and allowing participants control over their information through a self-administered questionnaire. For anonymity, participants did not disclose their names on the questionnaire and confidentiality was guaranteed through storing data in a locked space and their freedom to participate and withdraw from the study was explained prior to signing consent forms.

### Study design and setting

A descriptive cross-sectional study design was used to assess KAP and to undertake hazardous waste characterisation, with the variables of concern being KAP and masses and volumes of waste generated. For KAP assessment, the Total Population sampling method was selected for respondents (*n* = 30), including technical laboratory workers, sample transporters, cleaners, and data clerks. A survey was conducted using a self-administered semi-structured questionnaire and an observational checklist. The questionnaire and checklist were created by the researchers and approved by the Institutional Review Board and the Laboratory Head/Manager prior to data collection. The questionnaire was distributed to consenting respondents and collected data pertaining to KAPs. The observational checklist recorded staff practices vis-à-vis the laboratory’s standard operating procedure (SOP) for waste management. A total of 20 observations were made on a convenience sampling basis on six technical staff on duty during three shifts and two cleaners during two shifts to assess the implementation of HWM.

To enumerate waste generation, the waste stream was defined as all solid and liquid waste resulting from the processing of specimens. All waste generated on weekdays between the dates 16 May 2023 and 26 May 2023 was included in the study. An electronic balance was used to weigh the masses of solid waste, and estimation of chemical liquid waste volumes was undertaken using containers of known volume.

The laboratory is located approximately 105 km north-west of the capital, Maseru, and serves as a regional laboratory for referred specimens from nearby hospitals and selected health centres in the region. The laboratory includes a reception and six sections focusing on tuberculosis and coronavirus disease 2019 (COVID-19), molecular diagnostics, full blood count, CD4, microbiology, and clinical chemistry; all sections, including the reception, were included in the study assessments. On average, 13 625 specimens are processed monthly. Combustible risk waste emanating from tests is managed onsite through autoclaving and incineration, chemical waste is exported from the facility to the National Reference Laboratories for stockpiling and treatment, while general waste is collected and disposed of at the district’s designated disposal area by a contracted private company.

### Data analysis

For qualitative data, a category scheme was devised and data coded according to the categories. Descriptive statistics were then computed from the coded data and quantitative data using Microsoft Excel (2019) (Microsoft Corporation, Redmond, California, United States). Frequencies and percentages were used to express the data, from which a discussion was developed. Observations on KAP were made among the different categories of staff in the laboratory.

## Results

### Profile of respondents

The target population was *n* = 30 and a high response rate of 86.7% (26/30) was achieved through the consenting participants. Most respondents were women (53.8%), and 50% were aged 30–39 years (Table 1). Education level ranged from primary certificate to university postgraduate studies, where the majority (34.6%) had completed high school and college level. Work experience ranged from less than a year (15.4%) to a maximum of 20 or more years (11.5%), and the highest proportion of staff had 3–4 years of experience (26.9%).

**TABLE 1:** Socio-demographic characteristics of self-administered questionnaire respondents at a regional hospital, Lesotho, March 2023 to June 2023.

Socio-demographic characteristics	Variables	*n*	%
Position	Laboratory technologist	11	42.3
	Laboratory assistants	3	11.5
	Sample transporters	5	19.2
	Data clerks	3	11.5
	Cleaners	4	15.4
Sex	Male	12	46.2
	Female	14	53.8
Age (years)	18–29	3	11.5
	30–39	13	50.0
	40–49	6	23.1
	50–59	4	15.4
Work experience (years)	< 1	4	15.4
	1–2	2	7.7
	3–4	7	26.9
	5–9	5	19.2
	10–19	5	19.2
	≥ 20	3	11.5
Level of education	High school	9	34.6
	Vocational training	1	3.9
	College	9	34.6
	University undergraduate	4	15.4
	University postgraduate	1	3.8
	Other (Primary Certificate)	2	7.7

**Total**	**-**	**26**	**100.0**

### Knowledge of staff on healthcare waste management

**FIGURE 1:**
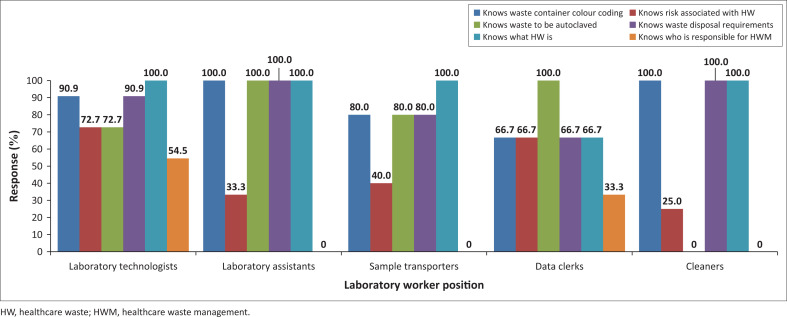
Healthcare waste management knowledge among laboratory workers at a regional hospital, Lesotho, March 2023 to June 2023. Included in the study were 11 laboratory technologists, 3 laboratory assistants, 5 sample transporters, 3 data clerks, and 4 cleaners.

All respondents (26/26; 100%) knew what HW is (Figure 1). In addition, all laboratory assistants (100%) answered three questions correctly, namely: the risk associated with HW, the colour coding used for segregating, and the requirements for HW disposal. Findings revealed that for three questions regarding what HW is, container colour coding, and disposal requirements, all 4 cleaners (100%) answered correctly, thus performing better than laboratory technicians, data clerks, and sample transporters on the latter two questions. A crucial finding also was that the question most poorly performed on by all cadres was regarding who is responsible for HWM in the laboratory, ranging from 0% among cleaners (0/4) to 55% (6/11) among laboratory technicians.

### Attitudes of staff on healthcare waste management

**FIGURE 2:**
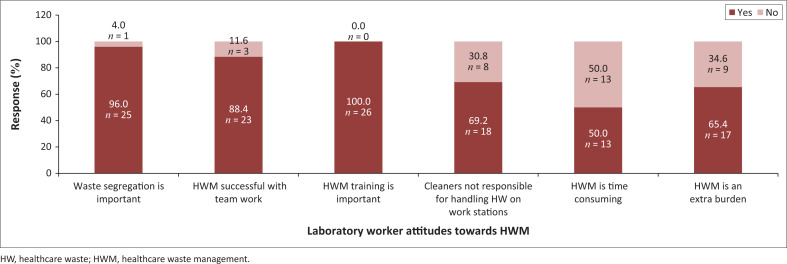
Laboratory workers attitudes towards healthcare waste management at a regional hospital, Lesotho, March 2023 to June 2023.

Ninety-six per cent (25/26) believe that segregation is key to HWM, while all 26 respondents (100%) agree on the importance of training to HWM (Figure 2). A positive attitude portrayed by 84% (22/26) of the respondents was that HW is best managed with teamwork. In addition, more respondents (69.2%; 18/26) felt that cleaners should not be allowed to handle all infectious waste on work benches. There was a tie at 50% (13/26) regarding whether HWM is or is not a time-consuming phenomenon. It was also discovered that 65.4% (17/25) of the respondents felt that HWM is an extra burden.

### Practices of staff on healthcare waste management

The laboratory has a waste management SOP which is in version 2 and was last reviewed on 07 February 2022 (Table 2). From the records, all technical and non-technical staff had signed that they had read and understood the SOP.

**TABLE 2:** Summary findings of healthcare waste management practices among laboratory staff at a regional hospital, Lesotho, March 2023 to June 2023.

Aspects of waste management and health and safety	Summary observations
Colour coding of containers	The colour coding and labelling were in accordance with the SOP and the Hazardous (Healthcare) Waste Management Regulations (2012).^28^ Containers were labelled according to the Hazardous (Healthcare) Waste Management Regulations (2012).^28^
Waste segregation	Five out of seven laboratory areas (71.4%) segregated waste according to the SOP. Microbiology and Molecular sections did not separate sharps from infectious waste. Sharps were contained in pathological waste containers.
Interim storage	The laboratory has a lockable interim storage outside the laboratory to temporarily hold laboratory waste only. Waste is stored for a maximum of 24 h. Waste is stored in an organised and clutter-free manner. The storage area is not locked at all times.
Onsite transportation	Laboratory waste is transported to the central incineration storage area using wheelie bins.
Treatment	Tuberculosis specimens and culture plates from microbiology are autoclaved. Autoclave tape is used as a validation method for the process. No records of the autoclave process/parameters are kept.
Handwashing after waste handling	None (0%) of the observed staff washed hands after handling waste.
Hepatitis vaccination	Six out of 27 (22.2%) of staff are fully immunised against Hepatitis while the rest were recorded as ‘not immune’.
Waste record-keeping and documentation	SOPs for waste management and use of the autoclave were kept. No records on amounts of waste generated, stored and autoclaved were kept.

Note: Please see the full reference list of this article for details on the article cited: Siimane TM, Nts’ihlele ME. Healthcare waste management knowledge, attitudes and practices of laboratory workers at a regional hospital, Lesotho. Afr J Lab Med. 2024;13(1), a2485. https://doi.org/10.4102/ajlm.v13i1.2485

SOP, standard operating procedure.

### Waste generated through laboratory practices

Out of seven (*n* = 7) days of segregation observations, the findings showed that the microbiology section did not comply with segregation requirements at all (0/7 days), while the tuberculosis and COVID-19 section complied for 4 out of 7 days (Table 3).

**TABLE 3:** Healthcare waste segregation compliance by work area at a regional hospital, Lesotho, 16 May 2023 to 26 May 2023.

Work area or section	Days with appropriate segregation	
	*n*	%
Reception	3	43
Molecular	2	29
Microbiology	0	0
Tuberculosis and COVID-19	4	57
Chemistry	1	14
CD4	1	14
FBC	2	29

COVID-19, coronavirus disease 2019; FBC, full blood count.

**FIGURE 3:**
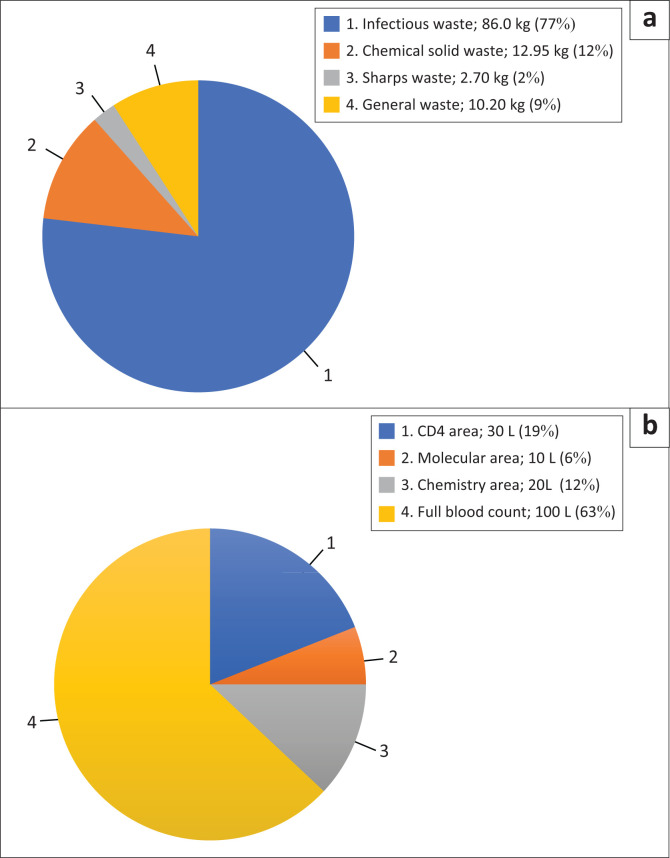
Characteristics of solid and liquid waste at a regional hospital, Lesotho, 16 May 2023 to 26 May 2023. (a) Solid healthcare waste and (b) liquid chemical healthcare waste not disposed to sewer.

Findings revealed that the laboratory does not routinely quantify and record the amounts of waste generated. For solid waste, the findings reveal that the category of HW generated the most is infectious waste (77%), while the lowest generated was sharps waste (7%) (Figure 3a). Healthcare general waste constituted 9% of the waste stream. The major infectious waste generating areas were the CD4, molecular, and tuberculosis and COVID-19 areas.

The majority of the chemical liquid waste that was not directly disposed of via the drain was generated by the full blood count area (63%), and the least emanated from the molecular section (6%) (Figure 3b).

## Discussion

This study, conducted between March 2023 and May 2023 at the regional hospital in Lesotho, found that laboratory workers have knowledge about HWM, and that workers’ attitudes towards HWM are predominantly positive. Despite the findings, some unsafe HW practices remain.

Demographic data show that the majority of participants were women (53.8%), which is in line with the country’s demographic profile, where women in the labour force constitute 53.5% in urban settlements.^30^ Love et al.,^31^ in a study conducted in the United States in 2022, concluded that a high proportion of women on scientific teams contributes to successful team collaboration, although it is not understood why this is the case or what proportion of women impacts team success. It is unclear whether the dominance of women in this setting influences KAPs in managing waste.

In general, it is known that the knowledge of workers will influence the attitudes and practices towards HWM.^32^ Participants who had no to low knowledge on who is responsible for HWM were the cleaning staff, sample transporters, and laboratory assistants. The lowest percentages regarding who is responsible for HWM were obtained from laboratory technicians (54.54%) and data clerks (33.33%). The lack of knowledge may lead to inefficiency of work, as duplication of efforts could lead to omission of work and lack of accountability. This aligns with the 2011 findings of a study undertaken in by Mathur et al.^8^ in India, attesting that inadequate and inappropriate knowledge of handling HW have serious health consequences and a significant impact on the environment. All cleaners (100%) answered correctly for the three questions regarding defining HW, container colour coding, and disposal requirements, thus performing better than laboratory technicians, data clerks, and sample transporters for these questions. Cleaners may therefore be further empowered as part of the laboratory’s support system to strengthen biosafety and biosecurity and continuous training can benefit all cadres in line with El-Gilhany et al.,^13^ whose study conducted at the Mansoura University, Egypt, in 2015–2016 showed that training brings significant improvement in waste management.

All participants (100%) had positive attitudes towards HWM training, and they were willing to take part in such trainings. The majority (88.5%) had a positive attitude towards managing HW as a team. The positive attitude could be harnessed by the laboratory to continue to champion improved HWM as demonstrated in a study conducted in South Africa in 2016 by Olaifa et al.,^25^ showing that there is a positive correlation between attitude and practice. On the other hand, there was high negativity (65.4%) of participants who found HWM as an extra burden proving the reluctance of participants to address the waste they generate. In their 2015 study in Pakistan, Khan et al.^11^ revealed similar findings among physicians and paramedic staff, identifying HWM as a financial burden.

The microbiology section indicated that it generated infectious waste only. There were no records indicating that pathological waste such as culture plates were autoclaved. This presents a biosafety and biosecurity challenge since it becomes impossible for the laboratory to account for the destruction of pathogens using validated methods as required by Chartier et al.^1^ and the United Nations Environment Programme.^33^ This could be attributed to the finding that some workers consider waste management to be a burden. In 2020, a study by Endris et al.^34^ established that medical laboratories in Addis Ababa, Ethiopia, properly performed waste treatment procedures. In 2011 in India, Mohankumar and Kottaiveeran^35^ observed the process of segregation, collection, transport, storage, and final disposal of infectious waste being done in compliance with the standard procedures. This is contrary to what was observed in some laboratory sections in this study, where requirements for segregation and containerisation of sharps waste were not performed according to the SOPs and national regulation. These practices expose waste handlers to sharps-related injuries. In consideration of the high levels of knowledge of containerisation among laboratory technologists (90.9%) and laboratory assistants (100%), this knowledge is not fully translated into practice, since some waste is contained in inappropriate colour containers. This differs slightly from the findings of where the majority (81.6%) of laboratory workers knew segregation requirements, and even more of them (91.8%) showed safe practices.^15^ Risk assessment and audits can be used to track such practices, and generally trainings can be followed up with studies that assess the effectiveness of training (on-the-job).

The laboratory generated more healthcare risk waste (91%) than healthcare general waste (9%). Of the healthcare risk waste, infectious waste was highest at 77%, and sharps waste the lowest (7%). This tallies with the 2018 findings of Mazloomi et al.^36^ in Iran, where infectious waste formed the majority, and sharps waste the minority. Most chemical liquid waste was generated by the full blood count area. The fact that the laboratory does not routinely quantify their waste is contrary to Radha et al.,^37^ whose 2009 study in India asserted that waste generated in a healthcare setting should be quantified to facilitate safe waste management systems. Waste quantification also can assist in adequate procurement and tracking of the use of waste containers. In addition, it guides the access to treatment facilities. The laboratory should consider quantifying and recording healthcare risk waste.

The full blood count area generated the majority (63%) of the liquid chemical waste, with the lowest amount emanating from the molecular area (6%). The chemical liquid waste is drained into an impermeable underground conservancy tank which is emptied by trained and authorised personnel. The tank contents are secured from unauthorised access, contrary to the findings of Ali et al.^2^ in their 2017 review, who establish that the majority of developing countries discard chemical waste from the laboratories into public sewers without any treatment. This act shows that the laboratory is ensuring biosafety and biosecurity precautions towards liquid waste generated.

It was observed that full hepatitis immunisation status was not achieved by the majority of workers (67.8%), which differs from the findings of Soyamet al.^19^ in their study conducted in India in 2011, where a significant proportion of workers (64.5%) were vaccinated. This practice compromises the occupational health and safety of laboratory personnel, and those exposed may develop laboratory-acquired infections. Training intervention on viral hepatitis has been shown to increase hepatitis vaccine uptake among laboratory staff and should be implemented.^13^

It was not clear as to when and how often temporary storage area was emptied of laboratory waste. Historically, HW was well secured and locked until recently, where the storage key was lost and the area left unlocked. In addition, the storage area has no signage to control entry, affirming some of the challenges of HWM as reviewed in 2017 by Ali et al.^2^ These conditions expose the waste to unauthorised persons and present a biosecurity risk. The study revealed that there are good HWM practices; however, some unsafe practices were also observed. Even though a clear and up-to-date SOP on HWM was available (review date 07 February 2022), there are some practices that are not conformed to. This is in line with the findings of the Ministry of Health.^38^ Additionally, knowledge and skills gaps that hamper SOP implementation could still exist among staff and should be identified and addressed.

### Limitations

Responses for the questionnaire were self-reported and could bring bias. Furthermore, because of the short period for monitoring waste generation, the picture of waste generation gained cannot be generalised over longer periods of operation. Lastly, inferential statistics could have improved the researchers’ understanding of the dynamics at play between KAPs.

### Conclusion

The laboratory has oversight mechanisms in place for engaging in safe HWM. While knowledge may exist among workers and attitudes may be positive, these do not always translate into the best practices for compliance with national regulation and SOPs. Therefore, improvement is needed to enhance biosafety and biosecurity. The study findings have been used to inform action such as refresher trainings, which are tied to monitoring and training impact assessment activities. Further investigation is needed to understand the factors leading to what hampers and promotes the observed attitudes and practices. As the laboratory continues to implement programmes that promote a culture of safety, performance monitoring should also be strengthened.
